# NT-pro-BNP as marker for cardiac strain that may be caused by high-output arteriovenous shunting in a haemodialysis patient. A case report

**DOI:** 10.1186/s12882-020-02195-9

**Published:** 2020-12-21

**Authors:** Michaela Wärja, Emelie Laveborn, Michael Ott, Andreas P. Jonsson, Bernd Stegmayr

**Affiliations:** Department of Public Health and Clinical Medicine, Umea University, SE 90187 Umea, Sweden

**Keywords:** Arteriovenous fistula, Congestive heart failure, Haemodialysis, NT-pro-BNP, Case report

## Abstract

**Background:**

An arteriovenous fistula (AVF) is the first choice when considering access for haemodialysis (HD). When a forearm AVF fails an upper arm AVF is a frequent subsequent dialysis access option. The latter may cause cardiac strain. NT-pro-B-type natriuretic peptide (NT-NT-proBNP) is a marker used to estimate volume overload and cardiac strain.

This case report shows the benefit of using longitudinal individual follow-up of pre-dialysis NT-proBNP in clinical practice to detect changes in cardiac condition that may be due to high-output AVF.

**Case presentation:**

An 18 years old patient performed HD via an upper arm AVF before he was admitted to our unit. NT-proBNP was above the upper detection level of 70,000 ng/L. Echocardiography revealed a left-ventricular cardiac insufficiency. Interdialytic weight gain (IDWG) was above 5%. He was instructed to lower fluid intake and IDWG towards 2%. Four months later NT-proBNP surpassed 70,000 ng/L again. Flow in the brachial artery was at 3034 ml/min. Reconstructive surgery of the AVF did not reduce flow and NT-proBNP in the long run. Clinically, he worsened to NYHA class III-IV. It was decided to close the upper arm AVF and to replace it with a lower arm AVF leading to a reduced artery flow of 1344 mL/min. The clinical condition successively recovered and NT-proBNP decreased to 7000 ng/L.

**Conclusions:**

Pre-dialysis NT-proBNP should be considered as a suitable routine marker for cardiac strain such as caused by high-output AVF besides variables such as IDWG. Brachial artery flow besides AVF flow measurement is helpful.

## Background

The optimal access for chronic haemodialysis (HD) is the placement of an arteriovenous fistula (AVF) in the forearm [1]. Stenoses and clotting may be restored by interventions [2], but still a substantial number of AVF cease to function. An alternative option is to place an arteriovenous graft (AVG). Over recent years AVG and upper arm AVF have become more frequent, especially in the USA and Europe [3]. However, the presence of a high-output AVF may stress the heart, particularly in patients with pre-existing heart disease [4]. Even the limited recirculation of blood to the heart in a lower arm AVF may cause some cardiac strain [5]. Since the proportion of upper arm AVF increases in numerous countries [3] the development of high-output heart failure may be an underappreciated complication [6–9]. When a large proportion of arterial blood is shunted from the left-sided circulation to the right-sided circulation via the fistula, the increase in preload can lead to increased cardiac output that over time may lead to cardiac hypertrophy and eventually congestive heart failure [10]. Patients may present with the classical signs of high-output heart failure such as tachycardia, elevated pulse pressure, hyperkinetic precordium, and jugular venous distension [6]. However, such symptoms may also develop due to myocardial injuries such as valvular defects or infarction. Another common reason for congestive heart failure is the retention of fluid between the dialyses. This volume overload, the interdialytic weight gain (IDWG) [11, 12], may even be hidden if the dry weight is lowered due to catabolism [12].

It is not always easy to be aware of an increase in cardiac strain induced by these factors. In our experience, the change over time in cardiac markers, such as the N-terminal prohormone of B type natriuretic peptide (NT-proBNP), helps to detect increased cardiac strain. NT-proBNP is a natriuretic peptide that is synthesised in the heart [13, 14]. A rise in NT-proBNP, is considered a response to stretching of the myocardial wall, what in normal circumstances will induce vasodilatation, natriuresis and diuresis [15, 16]. In patients with decreased kidney function and on dialysis NT-proBNP levels increase, partly due to decreased excretion [17, 18]. But, still NT-proBNP correlates with impaired cardiac function [19, 20]. These markers are also linked with increased risk of mortality [21–23]. The elevated levels of NT-proBNP in patients on dialysis have been explained not only by cardiac dysfunction/hypertrophy but also with volume overload [24, 25] such as large IDWG [11, 12]. However, in this case IDWG could not solely explain the worsened cardiac condition and progressing NT-proBNP data that indicated high-output AVF as a plausible reason.

The aim of this report was to show the benefit of using longitudinal individual follow-up of pre-dialysis NT-proBNP in clinical practice to detect cardiac strain that may be due to high-output AVF besides other variables such as IDWG.

## Case presentation

**Fig. 1:**
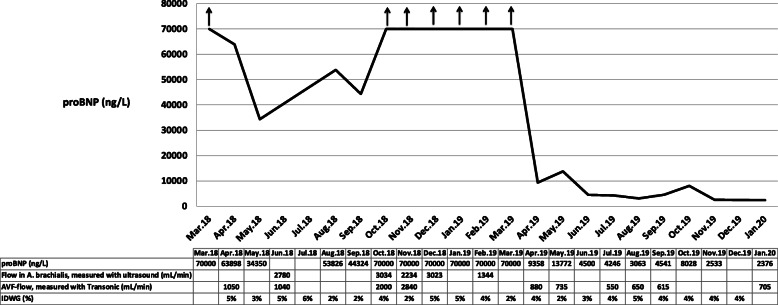
Distribution of NT-proBNP (proBNP), IDWG and arteriovenous fistula flow over time. Flow was measured (1) either in the brachial artery (A. brachialis) with duplex-sonography or (2) in the AVF (V. cephalica) and later lower arm AVF by Transonic® measurement. In Nov 2018, duplex-sonography and Transonic® measurements took place at different days

**Fig. 2:**
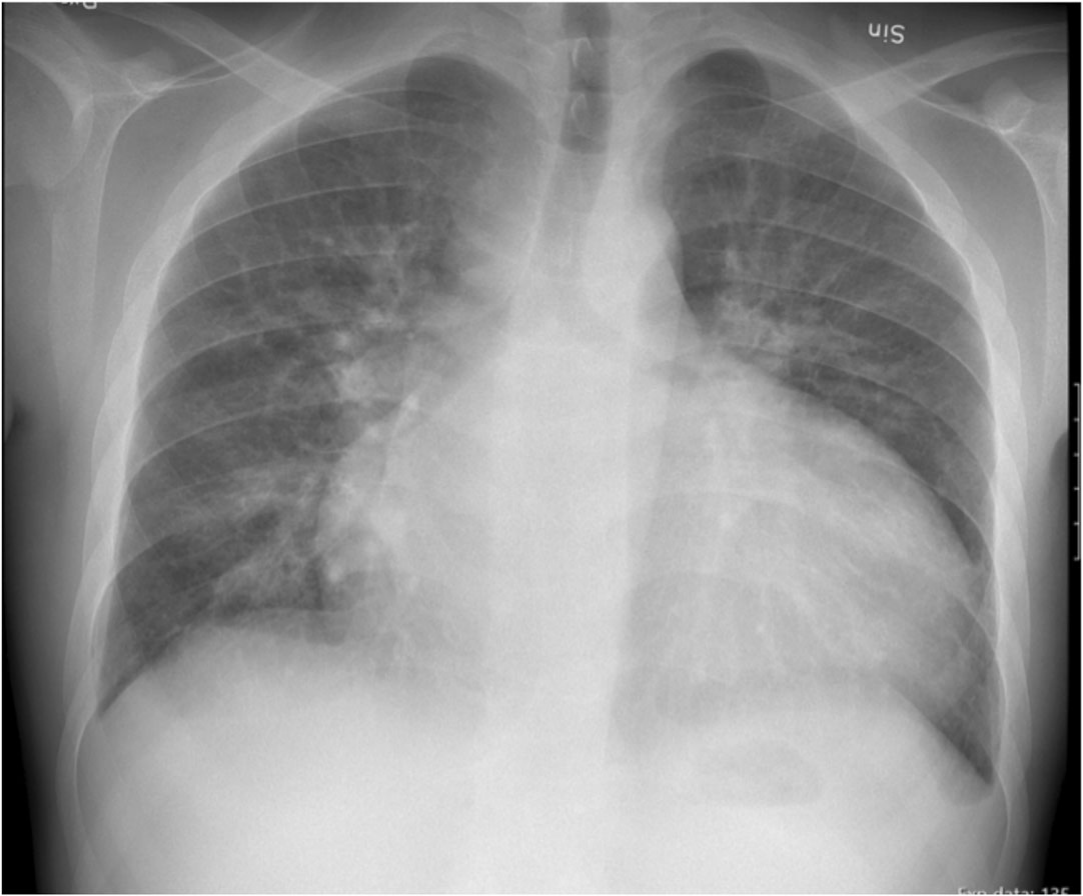
X-ray of the chest after HD visualizing a dilated heart

After written informed consent was achieved by the patient the following data is given: An 18-year-old man with an unknown cause of end stage renal disease started HD 3 years prior to this report. Initially, he had had an upper arm AVF on the left side. This AVF had to be closed due to a local invasive infection. Thus, an upper arm fistula was placed in the right arm and was thereafter used for HD. When arriving at our unit in March 2018, he suffered from intermittent pulmonary congestion that was clinically interpreted to be related to a high degree of IDWG caused by excessive fluid intake and water retention in conjunction with anuria. Body weight was estimated to be 60 kg. As shown in Fig. 1, the IDWG was approximately 5%. Intermittently, he had to perform additional acute ultrafiltration procedures since pre-dialysis NT-proBNP was above 70,000 ng/L (Fig. 1), and blood pressure around 160/105 mmHg. He was prescribed angiotensin receptor blockers and was told to strongly restrict fluid intake with the aim of IDWG towards 2%. He managed to limit IDWG to some extent, resulting in a transient lowering of NT-proBNP. After 5 months NT-proBNP surpassed 70,000 ng/L again. Chest X-ray showed pulmonary fluid retention and the heart size was extensively increased (Fig. 2). The patient suffered from progressive breathlessness during activity and was forced to access the dialysis ward by wheelchair; this corresponded to class III-IV of the NYHA functional classification [26]. From October 2018 until March 2019, NT-proBNP levels were above the upper limit of detection and considered a reflection of a severe cardiac strain. Ultrafiltration was increasingly difficult due to intradialytic hypotensive episodes. Blood pressure rose and doses of antihypertensives that included angiotensin receptor blockers, calcium-blockers and alpha-1 receptor antagonists had to be increased.

**Fig. 3:**
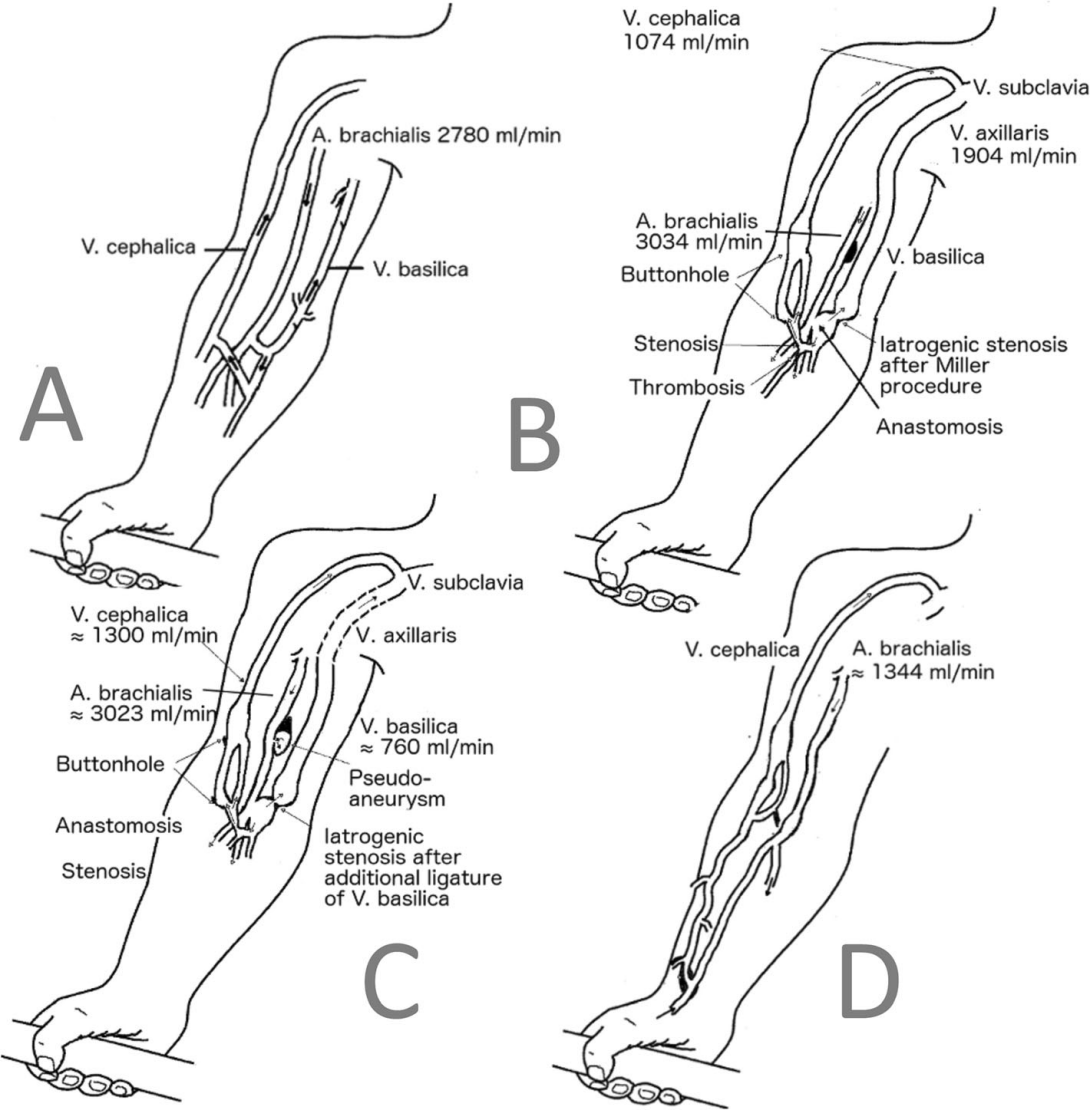
Schematic visualization based on duplex-sonography of vascular branches at the different stages of AVF. **a**: Baseline June 2018; **b**: After MILLER procedure Oct. 2018; c: After ligature of the basilic vein Dec 2018; **d**: after reconstruction Feb. 2019

Figure 3 illustrates the vascular conditions and flow measurements in the brachial artery as well as the flow through cephalic and basilic vein over time. While the patient had an upper arm fistula the flow in the cephalic vein was periodically surpassed by the flow through the basilica vein. In June 2018, the Doppler showed a flow of 2780 ml/min measured in the brachial artery representing 43% of the cardiac output.

Echocardiography showed a dilated left ventricle with reduced systolic function but a maintained stroke volume with an ejection fraction of 45% as shown in Table 1. A non-significant small pericardial effusion was seen. The extended QRS duration further emphasised the presence of dilated cardiomyopathy and heart failure [27]. Apart from what is mentioned in Table 1, the echocardiography showed structural changes, an atrial septum defect, and minor regurgitation of the mitral, tricuspid and pulmonary valves.

**Table 1:** Weight and cardiac findings at different time points

Measurements	Jun 2018	Feb 2019	Apr 2019	Jan 2020
Body weight, kg	60	60	58	70
Ejection fraction, %	45	42^a^	45	48
Stroke volume^b^, mL	72		63	93
QRS-duration, msec	136^c^	136	136	130

^a^In February 2019, a magnetic resonance investigation included measurement of ejection fraction. ^b^Stroke volume for the left ventricle measured at the aorta. ^c^ Sept 2018

In September 2018, a surgical correction according to Miller et al. [28] was performed. One month later partial ligation of the basilic vein was done. Within 1 month postoperatively brachial artery flow was back at 3023 ml/min despite a reduced diameter of 4 mm of the basilic vein (local flow 760 ml/min, Fig. 3c). Therefore, a temporary central venous catheter was placed, the upper AVF closed, and a new distal radiocephalic AV-fistula was successfully placed in the right arm in December 2018. Under the new radiocephalic AVF blood flow in the brachial artery decreased to approximately 1344 mL/minute when measured by ultrasound in February 2019. AVF flow measured by Transonic® was in the range of 550–880 ml/min. In March 2019, the dialysis sessions were increased from 3 to 4/week to limit the IDWG. In April 2019, echocardiography showed a slightly improved systolic function of the left ventricle (Table 1) but retained pericardial effusion. The NT-proBNP levels had dropped to 9358 ng/L. The patient still experienced breathlessness during activity, but it was less than before. No grading of the symptoms were registered. The echocardiographic findings and QRS duration improved at follow-up 8 months later (January 2020) and his clinical condition corresponded to NYHA I. Since then he felt well. After changing from upper arm to lower arm AVF, the AVF fistula flow (data by Transonic®) decreased as well as the NT-proBNP levels.

## Discussion and conclusion

The present study shows the additional prognostic value of analyses of pre-dialysis NT-proBNP as a routine individual marker in clinical practice. This young patient who initially lacked a history or clinical signs of cardiac problems, developed a life- threatening condition within 1 year of being on chronic HD. His impaired cardiac function was initially assumed to be solely attributed to high IDWG. However, reductions in IDWG only temporarily lowered the NT-proBNP and not as much as expected. Instead, the patient suffered from a progressive congestive heart failure (CHF). This forced us to re-evaluate the AVF as another reason for CHF and consider AVF surgery since large blood volumes shunted through the upper arm AVF. The condition improved first after a patent lower arm AVF was achieved and 4 dialyses/week initiated, which indicated the importance of limiting the return flow besides reducing the IDWG. Notably, such return flow should take into consideration the body weight of the patient. The European recommendation of AVF flow is to keep AVF flow above 600 ml/min, whereas the Japanese recommendation is to keep the flow between 500 and 1000 ml/min [12]. Data by Hadimeri et al. [5] indicate that even lower levels of blood return must be considered as a risk for heart strain, especially in vulnerable patients. The present case shows the importance of taking the brachial artery flow into consideration and not only the AVF flow. This is especially important if large anastomoses exist. In our case, a large return flow through the basilic vein exceeded the AVF flow (via cephalic vein) by far (shown in Fig. 3b). The return flow has to be put in relation to the size of the patient and the total cardiac output [29]. The present case supports repeated routine measurement of pre-dialysis NT-proBNP as it may help to detect progressive cardiac strain better and relate it to AVF flow changes. A rising NT-proBNP unexplained by the AVF flow should motivate measurement of brachial artery flow and screening for concurrent return flow by other veins. Fistula flow levels in the lower range should be accepted as far as they are sufficient enough to allow optimal HD.

A limitation of the study is that AVF flow was measured by Transonic® ultrasound dilution technique between dialysis needles. These lack brachial artery flow data which require the use of doppler sonography. In our case only one arterial flow was measured after the distal AVF was placed. The increase in frequency of dialyses from 3 to 4 /week may have reduced IDWG as another factor for cardiac strain. However, the limitation in improvement of IDWG over time strengthen the importance of the upper arm high-output shunt of the AVF. The strengths of the study are the repeatedly high NT-proBNP levels that motivated tight surveillance of vascular conditions in the arm in parallel with cardiac variables.

Routine access flow monitoring helps to demonstrate development of high-output cases and blood flow surpassing 1500 ML/min [6, 30]. Recommended is early ultrasound monitoring [31] and compression of the brachial artery while performing echocardiography [8].

Proximal radial artery ligation is an effective technique for distal radio-cephalic AVF flow reduction [30] and ultrasound guidance during intervention is helpful [32].

If congestive heart failure is already present before placement of an AVF one may question the necessity of an AVF and consider a central dialysis catheter (CDC) instead. The use of a CDC may not impair prognosis significantly in elderly when transplantation is not an option and long-term prognosis limited [33].

In conclusion this report emphasizes the benefit of routine measurement of pre-dialysis NT-proBNP to facilitate awareness of cardiac strain that may be caused by high-output AVF flow (measured by brachial artery flow) besides other variables such as IDWG.

## Data Availability

Data sharing is not applicable to this article as no datasets were generated or analysed during the current study besides Table and Figure included.

## Abbreviations

*AVF*: Arteriovenous fistula
*AVG*: Arteriovenous graft
*BNP*: B-type natriuretic peptide (BNP)
*CDC*: Tunnelled central dialysis catheter
*CHF*: Congestive heart failure
*HD*: Haemodialysis
*IDWG*: Inter dialytic weight gain
*NT-proBNP*: NT-pro-B-type natriuretic peptide (NT-proBNP), sample taken pre-dialysis
